# Factfinders for patient safety: Antibiotics for disc access and spinal cord stimulation trials

**DOI:** 10.1016/j.inpm.2022.100150

**Published:** 2022-11-25

**Authors:** Ryan Mattie, Byron J. Schneider, David C. Miller, Adrian Popescu, Clark C. Smith, Zachary L. McCormick, the Spine Intervention Society's Patient Safety Committee

**Affiliations:** a Total Spine Institute, Los Angeles, CA, USA; b Vanderbilt University Medical Center, Dept of Physical Medicine & Rehabilitation, Nashville, TN, USA; c Vanderbilt University Medical Center, Center for Musculoskeletal Research, Nashville, TN, USA; d Q C Kinetix, Denver, CO, USA; e Hospital of University of Pennsylvania, Department of Physical Medicine and Rehabilitation, Philadelphia, PA, USA; f Columbia University Medical Center, Rehabilitation and Regenerative Medicine, New York, NY, USA; g University of Utah School of Medicine, Department of Physical Medicine & Rehabilitation, Salt Lake City, UT, USA

## Abstract

This series of FactFinders presents a brief summary of the evidence and outlines recommendations regarding the use of antibiotics for disc access and spinal cord stimulation trials.

The evidence in support of the following facts is presented: (1) There is a low but nonzero risk of discitis due to percutaneous intervertebral disc access. Strategies to mitigate this risk include use of strict aseptic technique, use of a needle stylet, and prophylactic intravenous or intra-discal antibiotics. (2) In low-risk patients, it may not be necessary to continue antibiotics throughout the percutaneous or staged trial period; however, in high-risk patients, or in trials lasting more than five days, antibiotics should be considered on a case-by-case basis.

**Antibiotics for Disc Access**

Byron J. Schneider, MD; Adrian Popescu, MD; Clark C. Smith, MD, MPH; and Zachary L. McCormick, MD on behalf of the Spine Intervention Society's Patient Safety Committee.

**Myth: The risks of discitis from percutaneous intervertebral disc access and the role of prophylactic antibiotics are clearly defined.**

**Fact: There is a low but non-zero risk of discitis due to percutaneous intervertebral disc access. Strategies to mitigate this risk include use of strict aseptic technique, use of a needle stylet, and prophylactic intravenous or intra-discal antibiotics.**

*Incidence of discitis following intentional intra-discal access:*

The overall incidence of discitis following intentional percutaneous intra-discal access (“disc access”) in the absence of prophylactic antibiotics has been reported to be 0.24% [1]. The majority of available literature is from the 1980's and 1990's. Literature that reports 0% incidence of discitis following intentional disc access in the absence of prophylactic antibiotics is largely limited to small cohorts. In 1989, Jackson et al. reported 0 cases out of 124 patients and 231 discs accessed (herein referred to as n ​= ​124; 231) [2]. Notably, this study utilized a transdural approach to the disc. A 2003 study by Willems et al. reported an incidence of 0/200; 435 cases of discitis [1]. Conversely, other literature reports an incidence of discitis between 0.14% and 1.9% following intentional disc access without prophylactic antibiotics [[3], [4], [5]]. For brevity, only published manuscripts from 1980 onwards are reviewed here. In 1982, Milette and Melanson reported an incidence of 3/500; 1009 cases of discitis [3]. They cited “improper needle cleaning and rinsing prior to sterilization” as the cause, indicative of a likely dated technique and inadequate aseptic precautions [3]. In 1991, Simmons et al. reported an incidence of 1/164; 465 cases of discitis. While it is clear that prophylactic antibiotics were not used in the cohorts, other details are limited [4]. Most reports predominantly include lumbar disc access, but in 1980 Distelmaier et al. reported an approximate incidence of discitis of 3/800 (0.6%) cervical discography cases [5]. Guyer et al. (1997) reported on another cohort of patients who underwent cervical discography, reporting an incidence of 2/161; 269 (1.2%; 0.74%); in one case, *Staphylococcus epidermidis* grew from the biopsy culture [6]. One of the cervical discography cases ultimately resulted in quadriplegia [6].

The available literature regarding clinical presentation and risk of discitis following intentional disc access is limited to a small number of sentinel articles, which are detailed below:

*Clinical presentation of discitis following intentional intra-discal access:*

Guyer et al. described the clinical presentation of post-procedure discitis, based on the course of nine known cases [7]. The most common presentation was increased neck or lumbar axial pain (average time from procedure 17 days, range 3–48 days). An elevated erythrocyte sedimentation rate (ESR) occurred post-procedure on day 20 on average (range 30–116 ​mm/h with most cases above 60 ​mm/h). Positive bone scan findings were apparent by post-procedure day 33 on average; 7/9 bone scans performed on post-procedure day 18 were still negative. Two positive cultures were obtained, both *Staphylococcus epidermidis*. In this study, Guyer et al. also reported on a cohort of 2/2014; 6042 cases of discitis [7]. However, the additional nine cases of discitis described were not part of the cohort. This highlights that due to the relatively low risk of discitis, isolated cases may not be captured in small or medium-size cohort studies.

*Pathogenesis of discitis following intentional intra-discal access:*

Fraser et al. isolated bacteria from the disc in three of four patients with discitis who had open biopsies less than six weeks post discography and detected *pseudomonas aeruginosa*, *Staphylococcus epidermidis*, and *klebsiella pneumoniae* [8]. This, in conjunction with the above report by Guyer et al., suggests that discitis following intradiscal injection is caused by the introduction of bacteria present on the skin via the needle. Corroborating this theory, Fraser et al. noted an initial incidence of discitis of 6/222; 463 when using an open needle technique (needle without stylet), which equates to the highest reported rate of discitis in the published literature (2.7%; 1.3%). After changing to a two-needle technique and a styletted needle for disc access, the incidence of discitis decreased to 1/149; 283 (0.7%; 0.3%). In theory, passing a second needle that will penetrate this disc through the cannula of the first needle (that penetrated the skin) decreases the risk of seeding skin flora into the disc. However, it is unclear why, subsequent to this change, an additional 3/61; 134 (4.9%; 2.2%) cases of discitis were reported during the following two-years. While this one study suggests that the two-needle technique may reduce the incidence of discitis following discography, there is no corroborating evidence to support this.

*Antibiotic properties:*

Klessig et al. studied the properties of proposed prophylactic antibiotics with regard to bacteria thought to represent typical skin flora: *Escherichia coli B*, *Staphylococcus aureus*, and *Staphylococcus epidermidis* [9]. In an in animal in vitro model, IV cefazolin administered before discography was found to penetrate the intervertebral disc [9]. Intradiscal gentamicin, cefazolin, and clindamycin were found to remain efficacious in the presence of iodinated contrast. Minimum inhibitory concentrations were lower for cefazolin and gentamycin than for clindamycin. Interestingly, Iohexol alone also demonstrated some inhibition of cell growth [9].

In general, antibiotic coverage should address the typical skin flora thought to be causative. The most commonly cited recommendations for prophylactic antibiotics stem from expert opinion proposing options of cefazolin 1–2 ​g IV 30 ​min prior to discography or cefazolin 1–10 ​mg/mL with intradiscal contrast [10]. Physicians should also consider allergies and possible colonization with methicillin resistant *Staphylococcus aureus*. Further guidance regarding appropriate bacterial coverage can be found at https://www.cdc.gov/hai/data/portal/AR-Patient-Safety-Portal.html.

*Use of prophylactic antibiotics:*

There are no head-to-head studies to determine the comparative prevalence of discitis with and without prophylactic antibiotics.

Osti et al. reported on one of the only cohorts of patients who received prophylactic antibiotics before intentional disc access [11]. They reported an incidence of discitis of 0/127; 133 when intra-discal cephazolin mixed with iodinated contrast was used (1 ​mg cephazolin:1 ​ml of contrast). Follow up in this cohort was conducted at a minimum of three weeks. This study concomitantly reported on a study in a sheep model that compared prophylactic intravenous (IV) antibiotics to intradiscal antibiotics; IV administration resulted in intradiscal penetration of the antibiotic, and there was no difference in the prophylactic effect between the two delivery methods. These investigators also found that when IV antibiotics were started one week after intentional intra-discal contamination and administered for 21 days, bacteria were still present within the disc when the sheep were sacrificed. This is the best published evidence that antibiotics administered after disc injection are not as effective as prophylactic antibiotics and may not provide clinical value.

Pobiel et al. reported on a large cohort of patients that captured different clinical practice patterns [12]. Patients from the early portion of the cohort did not receive prophylactic antibiotics and were contacted by telephone up to five days post-procedure. Regular use of prophylactic antibiotics was then instituted, and routine five-day follow-up was discontinued. The overall incidence of discitis was reported to be 0/2085; 5981 in the cervical spine, 0/1141; 3083 in the thoracic spine, and 2/12,634; 37,135 in the lumbar spine. Given the high procedural volume and the fact that follow-up occurred either only five days post-procedure or was not routinely performed, the accuracy of these data is unclear. Details of the two cases of confirmed discitis were provided: the first was a three-level lumbar discography procedure in which antibiotic was not used due to possible allergy. Provocation of the two lower disc levels produced typical, concordant low back pain and were treated with intradiscal steroid without complication, but *Staphylococcus epidermidis* discitis developed in the healthy control L3–4 disc. The second case involved a three-level lumbar discography procedure wherein intradiscal gentamycin was used instead of cefazolin because of allergy to both penicillins and cephalosporins. Six-weeks post-procedure, the patient presented with worsening back pain, “somewhat different from her chronic pain” and a disc biopsy was inconclusive [12]. If the latter was indeed a case of discitis, it would be the only such captured in a cohort in which prophylactic antibiotics were used.

*The unknown:*

The available literature almost exclusively pertains to intentional disc access for the purpose of discography. It is unclear how these data pertain to disc access for other reasons.

Specifically, when disc access is inadvertent, such as during a transforaminal epidural steroid injection, it is likely that strict aseptic precautions were not present during the procedure, perhaps increasing the risk of infection. There is also no literature demonstrating that administering intradiscal, IV, or oral antibiotics after unintentional disc access prevents infection. This practice may be warranted to minimize the risk of infection given the morbidity associated with discitis but is not based on published evidence.

Similarly, the effect of prophylactic antibiotics during other procedures that include intentional disc access for therapeutic reasons, such as biologic treatments, has been sparsely reported in the literature. A case series of 15 patients undergoing intradiscal injection of fibrosealant fibrin reported a single case of post-injection discitis (*Haemophilus parainfluenzae* types 1 and 2, *alphahemolytic Streptococcus* sp., and nonpathogenic *Neisseria* sp.) [14]. There is a single report of discitis (*Cutibacterium acnes*) following L5-S1 intradiscal platelet rich plasma injection [15]. While the effects of antibiotics on the viability of the biologic products once inside a relatively avascular structure are unknown, these cases highlight the risk of potential discitis occurring with these procedures.

## Conclusions

⁃ The risk of discitis occurring after intentional disc access is non-zero, with reported rates ranging between 0.4 and 1.9% when performed in the absence of prophylactic antibiotics.
⁃ The literature suggests that if discitis occurs, it is likely due to bacteria commonly colonized on the skin.
⁃ The available literature suggests that this risk of discitis is lower when prophylactic antibiotics are used, but this is limited to only 2 cohort studies.
  o The risk may differ depending on cervical vs thoracic vs lumbar segments.
  o If prophylactic antibiotics are used, medications that cover typical gram-positive skin flora should be used.
    ⁃ Other resources suggest administration of Cefazolin 1–2 ​g IV 30 ​min prior to discography and/or cefazolin 1–10 ​mg/mL with intradiscal iodinated contrast.
⁃ There is no published literature pertinent to the use of antibiotic administration following inadvertent disc access. One must balance the low, but serious risk of potential discitis against the inherent risk with giving antibiotics.
⁃ Utilizing strict sterile/aseptic conditions and utilizing chlorhexidine solution prep according to the manufacturer recommendations are strongly recommended prior to disc access.

**Antibiotics for Spinal Cord Stimulation Trials**

Ryan Mattie, MD; David C. Miller, MD, MA; and Zachary L. McCormick, MD on behalf of the Spine Intervention Society's Patient Safety Committee.

**Myth #1: Patients undergoing spinal cord or dorsal root ganglion stimulation trials must be kept on prophylactic antibiotics throughout the entire course of either a percutaneous or staged trial while electrodes remain externalized.**

**Myth #2: Only patients undergoing a staged trial should be placed on prophylactic antibiotics during the trial period.**

**Fact: In low-risk patients, it may not be necessary to continue antibiotics throughout the percutaneous or staged trial period; however, in high-risk patients, or in trials lasting more than five days, antibiotics should be considered on a case-by-case basis.**

Spinal cord stimulation (SCS) and dorsal root ganglion (DRG) stimulation are evidence-based procedures used to treat a variety of chronic pain conditions. Although the electrodes that provide stimulation can be directly implanted, patients typically undergo a trial period of stimulation in order to provide confidence that implantation will result in pain and functional improvement [15]. There are two strategies for placement of electrodes during a trial, detailed below. In both cases electrodes remain externalized for the duration of the trial. This forms a potential conduit between the skin and the deeper tissue planes, which may inadvertently facilitate an infection. The literature poorly discriminates between infections following a trial period or infections following permanent implantation [16]. This has contributed to heterogeneous practice patterns surrounding antibiotic use during the trial period.

While there are general recommendations addressing antibiotic use in SCS, existing guidelines [16,17] do not adequately address its use in the trial period when leads are externalized. These guidelines recommend preoperative antibiotic prophylaxis be administered 30–60 ​min before insertion/incision with doses determined on the basis of patient weight. They advise against continuation of antibiotics beyond one dose for both neuromodulation trials and implants. An international survey of more than 500 physicians investigating infection control practices for SCS reported that about 50% of respondents continued antibiotics into the postoperative period [18]. Of the respondents that continued antibiotics post-operatively during the trial period, 90.2% (244) did so for more than 24 ​h. In this study, two factors cited for consideration when determining whether or not to continue antibiotics during the trial period were: 1) the type of trial performed, and 2) the anticipated duration of the trial.

*Trial Type*.

Multiple strategies exist for the trialing of neuromodulation devices, but the two most common consist of either a percutaneous trial followed by a separate full implant, or a staged trial and direct implant. In the first method, temporary electrodes are placed percutaneously and attached to an external pulse generator/battery for the duration of the trial. The temporary electrodes are removed at the end of the percutaneous trial. Subsequent implantation with permanent leads may be undertaken several weeks later. With a staged trial, the “trial” stimulating electrodes become the permanent electrodes and are anchored to the fascia before being connected to temporary extension leads that are tunneled to the region of the pulse generator/battery location and externalized for the trial period. This necessitates a more extensive surgical implantation procedure during the trial lead placement than if percutaneous leads are removed at the conclusion of the trial period. In the survey referenced above, 96.8% of the respondents from the United States reported using the percutaneous trial and implant pathway [18].

Previous concerns have been raised regarding the possibility of increased infection rates associated with the staged trial pathway, though the current literature often does not discriminate between the technique of SCS during the test period and the implantation when reporting infection rates [16]. May et al. reported on the first 59 SCS patients (1993–1997) undergoing staged trials, and 18.6% [95% confidence interval (CI): 9.7–90.9%; 11 patients] had an infection necessitating antibiotic therapy and a delay before permanent system implant [19]. Improvements in technique and wound dressing in a subsequent series reduced the trial infection rate to 7.5% (95% CI: 1.6–20.4%; 3 of 40 patients), with all of the infections being superficial. Rudiger et al. also evaluated staged trials and reported an infection rate of 1.2% (95% CI: 0–6.5%; 1 of 84 patients) [20]. A 2007 study of patients who underwent either a percutaneous or staged trial demonstrated similar results with only one infection in 65 SCS trials (1.5%, 95% CI: 0–8.3%), though the study did not specify if the infection developed with a percutaneous or staged trial [21].

Additionally, in a retrospective review of 286 patients comparing outcomes and adverse events between percutaneous and staged trials, Simopoulos et al. observed a lower infection rate in the percutaneous trial group (1.35%; 95% CI: 0.4%–2.3%; 2 of 148 patients) compared to the staged trial group (6.5%; 95% CI: 4.4%–8.6%; 9 of 138 patients), which was statistically significant (p ​= ​0.02) [22]. Conversely, a large, multicenter, retrospective observational analysis of 2737 SCS implants or revisions by Hoelzer et al. found that permanent implants after a staged trial had an infection rate of 0.9% (95% CI: 0.4–1.9%; 7 of 753 patients), while permanent implant after a percutaneous trial had an infection rate of 2.1% (95% CI: 1.3–2.9%; 27 of 1308 patients) [23].

*Trial Duration*.

Many key SCS efficacy studies have relied on an extended trial period as part of their selection criteria [21,24,25]. In a review of 84 patients with SCS implantations, Rudiger et al. reported an average screening trial period of nine days, with a range between four and 29 days [20]. In the review above by Hoelzer et al., the average trial length was 4.8 days (no range was provided) [23]. The same review also found that patients who underwent permanent implantation of an SCS system after a previous trial lasting more than five days had a significantly higher risk of infection than those whose trial was five days or fewer (3.7% and 1.6%, p ​= ​0.05). Importantly, these infections affected the permanent implant and not the trial itself. A common assumption that a longer trial provides greater opportunity for infection to develop anywhere between the skin and the spinal canal may be reflected by these data.

In another retrospective review of 707 SCS cases, Mekhail et al. suggested that an adequate trial lasting from five to 14 days is required for appropriate patient selection, as SCS implantation in poorly selected patients may be potentially harmful and costly [26]. This review reported an SCS-related infection rate of 4.5% (95% CI 3.1–6.3; n ​= ​32) but did not specify if these infections were trial or implant related.

*Comorbidities*.

It has been established that certain comorbidities – including tobacco use, uncontrolled diabetes, malignancy, human immunodeficiency virus (HIV), untreated remote infections, *Staphylococcus aureus* carriers, and preoperative steroid use – may result in a greater risk of infections in other surgical arenas [[27], [28], [29], [30], [31]]. But the question of whether or not this increased risk exists throughout the SCS trial period is unclear. In the retrospective review by Hoelzer et al., no statistically significant differences in SSI rate with obesity, diabetes, or tobacco use were identified, but it should be noted that the data are referring to SCS implants, and not the trials [23]. Additionally, in this same study, 2024 out of 2586 cases (78.4%) received post-operative antibiotics with an infection rate of 1.78% (n ​= ​36), while patients not receiving post-operative antibiotics had an infection rate of 4.09% (n ​= ​23, p ​= ​0.001). Individual data on those patients with comorbidities receiving post-operative antibiotics versus those with comorbidities who did not receive post-operative antibiotics are not available. The average duration of prescribed post-operative antibiotics was 7.6 days. Notably, in the study by Mekhail et al., patients with diabetes and failed back surgical syndrome (FBSS) showed increased rates of infection (9% and 6.3%, respectively) beyond the average incidence of 4.5% with implantation, but neither was found to be statistically significant [26]. No infections were documented in any of the 707 SCS trials, but the authors did not disclose the antibiotic regimen used during the trial period.

*Spinal Epidural Abscess*.

Perhaps the most concerning complication related to SCS or DRG lead placement is the potential development of a spinal epidural abscess (SEA). The retrospective study by Mekhail et al. reported 18 cases of SCS-related SEAs, but none apparently occurred during the trial [26]. There are only two peer-reviewed case reports of SEA developing specifically during an SCS trial. The first case in 2008 involved a 46-year-old male, smoker with FBSS, who underwent placement of percutaneous leads without receiving any pre- or post-procedure antibiotics. The SEA became apparent on post-op day five [32]. In the second case, a 54-year-old healthy female with lumbar radiculopathy underwent a percutaneous trial with 2 ​g of intravenous cephazolin for prophylaxis and was discharged with no additional antibiotics [33]. On trial day four, her leads were removed and oral antibiotics were initiated because of fever. SEA was discovered on post-op day five.

## Conclusions and Recommendations

1) SCS and DRG stimulation trials have a reported infection rate of 1–18% [16,[18], [19], [20], [21], [22], [23], [24]]. Significant reductions in infection rates have been achieved during the past decade. In the largest contemporary evaluation of SCS-related infections, Hoelzer et al. calculated an infection rate of 2.45%, which is comparable to the general incidence of surgical site infections across multiple surgical specialties [23,34].
2) It remains undetermined whether percutaneous or staged stimulator trials have a higher infection rate, as studies have shown conflicting results.
3) All appropriate measures should be taken to limit infection risk during SCS and DRG stimulation trials, including strict sterile technique during lead placement, exit-site dressing management with hydrocolloid and occlusive dressing, and adherence to the perioperative antibiotic regimen as outlined in the *SIS Safety Practices for Spinal Cord and Dorsal Root Ganglion Stimulation* and existing guidelines [16,35].
4) While current recommendations for surgical site infection prevention include administration of antibiotics prior to surgical incision and at most for 24 ​h following surgery [16,17], there may be instances in which post-operative antibiotics beyond 24 ​h are indicated during an SCS or DRG stimulation trial.
  A) Data demonstrate that patients with comorbid diseases such as those with diabetes, malignancy, tobacco use disorder, remote infections, steroid exposure, *Staphylococcus aureus* carriers, and those who have undergone a prior spine surgery, have an increased risk of infection associated with surgical implantations [26], though this relationship has not been shown specifically in SCS trials.
  B) Trials lasting more than five days have been associated with higher infection rates for the subsequent permanent implant [23].
  For patients undergoing planned SCS or DRG trial for a duration greater than five days, or for those with certain comorbid diseases that may predispose to an increased risk of infection, administration of prophylactic antibiotics throughout the course of the trial should be considered. However, it must also be acknowledged that increased duration of antibiotics increases adverse drug events such as development of *Clostridioides difficile* and postoperative infection due to drug-resistant organisms [36].

5) Even with the appropriate pre-operative, intra-operative, and post-operative antibiotic prophylaxis in an otherwise healthy patient, development of an SEA remains a serious concern with SCS and DRG stimulation devices, and there should be a low threshold for repeating a spinal MRI if complications develop.

## Declaration of competing interest

The authors declare that they have no known competing financial interests or personal relationships that could have appeared to influence the work reported in this paper.
